# Advancing healthcare practice and education via data sharing: demonstrating the utility of open data by training an artificial intelligence model to assess cardiopulmonary resuscitation skills

**DOI:** 10.1007/s10459-024-10369-5

**Published:** 2024-09-09

**Authors:** Merryn D. Constable, Francis Xiatian Zhang, Tony Conner, Daniel Monk, Jason Rajsic, Claire Ford, Laura Jillian Park, Alan Platt, Debra Porteous, Lawrence Grierson, Hubert P. H. Shum

**Affiliations:** 1 Department of Psychology, Northumbria University, Northumberland Building, College Lane, Newcastle Upon Tyne, NE1 8SG UK; 2 Department of Computer Science, Durham University, Durham, UK; 3 Department of Nursing and Midwifery, Northumbria University, Newcastle Upon Tyne, UK; 4 Department of Family Medicine, McMaster University, Hamilton, Canada

**Keywords:** Healthcare professional skills, Nursing skills, Competency-based education, Deep learning, Pose estimation, Healthcare skills databases

## Abstract

Health professional education stands to gain substantially from collective efforts toward building video databases of skill performances in both real and simulated settings. An accessible resource of videos that demonstrate an array of performances – both good and bad—provides an opportunity for interdisciplinary research collaborations that can advance our understanding of movement that reflects technical expertise, support educational tool development, and facilitate assessment practices. In this paper we raise important ethical and legal considerations when building and sharing health professions education data. Collective data sharing may produce new knowledge and tools to support healthcare professional education. We demonstrate the utility of a data-sharing culture by providing and leveraging a database of cardio-pulmonary resuscitation (CPR) performances that vary in quality. The CPR skills performance database (collected for the purpose of this research, hosted at UK Data Service’s ReShare Repository) contains videos from 40 participants recorded from 6 different angles, allowing for 3D reconstruction for movement analysis. The video footage is accompanied by quality ratings from 2 experts, participants’ self-reported confidence and frequency of performing CPR, and the demographics of the participants. From this data, we present an Automatic Clinical Assessment tool for Basic Life Support that uses pose estimation to determine the spatial location of the participant’s movements during CPR and a deep learning network that assesses the performance quality.

**Supplementary Information:**

The online version contains supplementary material available at 10.1007/s10459-024-10369-5.

## Introduction

Healthcare professionals, across all disciplines, must master many movement-based technical skills to ensure positive outcomes for patients and avoid injury to themselves. Improper patient lifting techniques can cause harm to both patient and practitioner; inaccurate intubation can damage vocal cords, and inefficient surgeries are linked with poor operative results. Due to the importance of quality care, training programs for healthcare professionals around the world have started to shift curricula towards an education paradigm known as competency-based education (CBE), which eschews time-based schedules of learner progression in favour of systems of matriculation that depend on direct observation of learner competence across a pre-defined set of professionally relevant activities (Frank et al., 2010; Harden, 2007).

The subjective nature of competency-based assessment provides a key challenge. Where some healthcare disciplines [e.g. robotic-assisted surgery (El-Sayed et al., 2024)] have devoted more research toward understanding how objective measurements of movement relate to expertise and the clinical outcomes of patients, most disciplines have lacked the technology to easily extract such information to determine how and when movement patterns matter for the patient and practitioner. Innovations in computer science that provide an opportunity to study technical skills in simulated and real environments without needing specialised recording devices provide this much-needed opportunity for healthcare skills more generally. Thus, empirically validated competency targets could be established by analysing video datasets that can be easily accumulated during training and practice across multiple healthcare disciplines.

A second challenge of CBE is that it is human resource intensive. CBE requires frequent formative and summative assessments to be implemented by healthcare educators who may have contemporaneous patient care and educational commitments. With appropriate objective competency thresholds established, automated feedback and assessment systems could provide the opportunity for self-directed deliberate practice, lessening the amount of formative feedback required from a coach and allowing students who need more practice time to have that opportunity. These formative assessment techniques could also flag when someone may meet the required competency and is ready to be assessed for progression. Such tools would also represent cost savings given that they would reduce the human resource requirements of training, which is of rising concern within the educational sector (Castillo et al., 2019).

Yet, the above benefits cannot be achieved without substantial investment in accumulating and sharing relevant data. Thus, this paper represents a call for collective effort from healthcare institutions (both educational and providing) toward amassing skills performance data that can be used to determine clear competency thresholds that healthcare students, educators, and professionals can target to enhance patient and practitioner outcomes. With recent innovations, the computational barrier to processing and analysing such complex data no longer exists; instead, the barrier is access. The surgical field has a much longer history of evaluating human movement-based skills on a kinematic level and has begun to look at implementing pose estimation and artificial intelligence techniques to better understand surgical skill and enhance education (Constable et al, 2024a; Likosky et al, 2021). As such, surgical tool and procedure datasets (Bouget et al., 2017; Srivastav et al., 2018) are currently being collated to accelerate the development of specialised pose estimation algorithms within the discipline. We suggest that all healthcare disciplines could benefit from such an agenda and call for a concerted effort across healthcare and science, more broadly, to develop policies and practices that provide the means to develop technologies to support healthcare trainees, professionals and patients alike.

By creating repositories of skills performance data alongside other factors of importance (e.g. demographics, educational level, patient factors), collaborative efforts from researchers in the fields of computer science, data science, human movement and healthcare education can begin to meet the first challenge of establishing objective, validated, and measurable competency-based thresholds. Furthermore, established competency thresholds and videos of skills performances will subsequently assist computer scientists in building rigorous skills assessment tools. With the two above challenges in mind, the present work aims to demonstrate how the accumulation of skills performance data sets can be combined with innovations in computer science (specifically, pose estimation and deep learning for the classification of expertise) to study technical competencies within healthcare professional education and provide automated means of assessing skills performance for the educational setting.

In computer vision, pose estimation refers to tracking movement from videos or pictures. Here, we focus on deep learning techniques which involve ‘training’ an Artificial Neural Network (ANN) on annotated videos or stills that indicate the relevant objects or body parts to be tracked (Ionescu et al., 2014; Lin et al., 2014). After training, the ANN can identify the relevant body parts or objects and, in turn, ‘poses’ within videos without human assistance. The kinematic parameters of a given movement can be calculated from body part locations and the frame rate of the video. Although these techniques require more powerful hardware than a standard computer, acquiring and setting up such tracking is more accessible than using specialised motion tracking cameras because it can be done with traditional video cameras. Further, pose estimation algorithms and toolboxes are advancing rapidly, with multi-person pose estimation possible with pre-trained networks at near real-time speeds (Huang et al., 2020), meaning that real-time feedback could be provided.

The kinematic data obtained from pose estimation can be used to levy an assessment of movement or to support formative development. The kinematic data could be used directly or by classifier algorithms to provide further assessment. For example, optimal posture during CPR requires the practitioner’s shoulders to be directly above the patient. Deviation from this optimal posture could be relayed back to the trainee to allow them to explore and feel their approach. Pose estimation data could complement instructor observations or data from computerised manikins, allowing trainees to refine psychomotor techniques, improving compressions and protecting practitioners from injury. Such applications of kinematic feedback have been repeatedly demonstrated in high-stakes sports training environments (Giblin et al., 2016; Glazier, 2021) and surgical training environments (Judkins et al., 2008) and are consistent with fundamental teaching and learning theory (Ericsson, 2004; Platt et al., 2021).

Classifier algorithms can extend on the above by making decisions along a given dimension. Here, neural networks are trained on relevant data that could be used to decide on competence (e.g., pose estimation data and evaluation data). Classifier algorithms have been demonstrated as useful and highly accurate for both assessment purposes and for highlighting aspects of the skill for improvement (e.g. suturing, (Ismail Fawaz et al., 2018)). For example, if competency is of interest, the ANN must be trained in example performances to learn patterns representing good, adequate, or poor performance. The neural network can then identify the competency level displayed in new videos based on learned patterns. Nevertheless, it is vital to be cautious in the development of these algorithms: the decisions risk being biased if the training data does not accurately represent the to-be-assessed data or the intended purpose (Veale & Binns, 2017); decisions may be based on parameters that co-occur with the classified groups but are not meaningful to the decision-making process. Considering the quality, depth and breadth of the training data can assist in protecting against such concerns.

While such tools are more commonly applied directly to tasks that are weighted toward psychomotor ability, these tools could also be used to assess non-technical skills. For example, situational awareness is critical in many healthcare contexts but is challenging to teach and assess. Simulation-based educational interventions yield better outcomes in situational awareness training (Walshe et al., 2019). In high-fidelity clinical simulations, final-year nursing and paramedicine students perceived that using eye-tracking technology combined with video debriefing assisted in their development of situational awareness (O'Meara et al., 2015). This intervention required participants to wear eye-tracking glasses, which may be challenging to implement for many training programmes. However, recent advances in pose estimation show that human attention can be tracked and modelled within a task space with information about head pose and orientation. Of course, this is less precise than eye-tracking. Nevertheless, this technique has been demonstrated to be a viable method of assessing concentration loss, collaborative attention and stress levels more generally for industrial applications (Lagomarsino et al., 2022), suggesting that pose estimation techniques could assist in the tracking and understanding situational awareness for healthcare applications.

### Considerations in implementing pose estimation and classifier algorithms

Such techniques have limitations, especially in real healthcare settings, which are often busy and complex environments. It is possible that occluded points will not be estimated or will be estimated with lower accuracy. Using multiple cameras (Kocabas et al., 2019) will alleviate this issue. A range of gap-filling strategies, including those employing ANNs (Kanazawa et al., 2018), can also be used to estimate missing data. If high precision is still needed, a hybrid approach could be used with specialist simulators combined with a visual approach. For example, manikins that track compression depth and rate (e.g. QPCR manikins from Laerdal) could be used simultaneously with video data, which allows posture to be tracked. With further advances in computer vision techniques, accuracy thresholds across all relevant dimensions may reach a level where a hybrid approach may not be needed for high-precision cases.

Just as humans make mistakes, algorithms can too. A recent systematic review evaluating the use of machine learning for classifying surgical expertise indicated typical accuracy rates of over 80% (Lam et al., 2022). Accuracy rates are likely to rapidly improve as appropriate video data is obtained for development purposes; nevertheless, even with accuracy rates at 80%, trust and acceptance of the use of classifier algorithms for assessment would be enhanced with a ‘human-in-the-loop’ approach (Enarsson et al., 2022) that provides a means of cross-checking. With human oversight, classifier algorithms could be used to track progression against milestones for healthcare trainees and flag when a student is ready to be formally assessed against competency thresholds by a human assessor. Importantly, the human in the loop must be participatory; decisions should not blindly follow the classifier’s recommendation and decisions should be monitored if this strategy is used (Kazim et al., 2021).

Considering potential bias and how the algorithm makes decisions is ethically crucial in developing classifier algorithms. If the data used to train the algorithms is biased, then this bias will be evident in any decisions. This point highlights the importance of accessing wide and diverse data sets. Of course, it can be challenging to determine if a data set is biased for the purposes for which it is being used. Classifier algorithms that output a meaningful description of the decisional parameters [Explainable Artificial Intelligence, (Taylor & Taylor, 2020)], combined with a human-in-the-loop approach, can assist in mitigating any potential bias. Unfortunately, classifier algorithms often disadvantage underrepresented groups (Holstein et al., 2019). Thus, carefully considering the training data is essential to ensure the algorithms’ fair and equitable decisions (Corbett-Davies et al., 2023; Veale & Binns, 2017).

In some cases, it may be reasonable to develop algorithms that ignore protected characteristics to ensure that the algorithms cannot learn the systematic biases present in society; however, in many cases, this may eliminate important information which impacts conclusions made (Hajian & Domingo-Ferrer, 2013). For example, in the case of physical disability, movement patterns may be markedly different. Ignoring that characteristic of the performer may lead to improper classification of competence, particularly when they have found a viable movement pattern that deviates from the sample norm. If an algorithm considers such diverse information, then the outcome will likely be fairer (Veale & Binns, 2017). Fairness and equity will require careful consideration of the context. Ensuring that systematic discrimination is avoided during implementation must be a high priority (Hagendorff, 2019). For thorough reviews and strategies for anti-discrimination in machine learning, see Veale & Binns (2017) and Hagendorff (2019).

### Data acquisition, storage and use considerations

Beyond the demonstrated application, opening doors to movement analysis in real healthcare settings provides opportunities to understand how movement patterns relate to patient outcomes in a given environment through data mining. Such a pursuit could lead to data-driven support for change to environments and policies that support practitioners. Furthermore, it has been challenging in some healthcare professions to link competency milestones with patient outcomes (Kendrick et al., 2023). Large datasets that speak to the trajectory of expertise, patient outcomes, and practitioner injury would allow the profession to develop competency thresholds informed by empirical evidence where necessary. Yet, data availability is currently limited, and initiatives to accumulate and share such data require careful consideration.

Where the potential benefit of collecting and evaluating video performance data to enhance healthcare appears substantial, there are also significant barriers. Installing cameras to monitor patient care (e.g. operating room black boxes) is becoming more commonplace; however, healthcare professionals have raised concerns over data safety and litigation. Cultural factors seem to play a role in such concerns. For example, Canadian healthcare professionals are more concerned (Gordon et al., 2022) than Danish healthcare professionals, who indicated relatively high opinions toward monitoring initiatives (Strandbygaard et al., 2022). Where it is most certainly essential to consider acceptance to maintain an environment of trust, it is also important to note that regardless of perception, video data most often supports healthcare professionals from a legal standpoint (van Dalen et al., 2019) and thus is most likely to offer protection in a litigious environment.

Legal policies around recording healthcare professionals, students and patients will likely differ considerably between governing bodies. Still, there has been a considerable cultural shift toward prioritising the data privacy of individuals and ensuring that personal data is protected, with the General Data Protection Regulation (GDPR) being one of the most comprehensive examples globally. Within the (evolving) legal framework, institutions should develop strong policies to ensure that video footage and any associated data regarding outcomes is recorded, stored, and used ethically and legally.

Data minimisation is one principle that may arise in both legal and ethical frameworks globally (e.g. Europe’s GDPR and California’s CPRA) that allows for a balance between the processing of personal data and data privacy (Goldsteen et al., 2022). This principle requires that the minimum amount of data be collected and processed. However, this practice could hinder finding important patterns between clinical practice, patient and practitioner factors, and patient outcomes without careful consideration. Identifying patterns that result in incremental gains to patient outcomes is important in healthcare situations. Where identifying patterns in such large datasets may not have been previously possible, machine learning opens the doors to such pursuits, and the importance of a particular variable may not be known ahead of time.

‘Big Data’ seems at odds with principles of data minimisation, and indeed, recently the UK’s Information Commissioner’s Office has indicated that data minimisation should be applied at both training and inference stages of machine learning (Kazim et al., 2021) with global regulatory bodies also providing guidelines for ethical use. In response, new methods are being developed to minimise the data required whilst maintaining accuracy or providing evidence that the minimum amount of data was used to achieve the aims (Goldsteen et al., 2022). As the use of artificial intelligence technology becomes more common, discussion of how to balance data privacy with scientific advancement will likely become a particularly hot topic from an ethical standpoint. Regardless, adherence to the Declaration of Helsinki (World Medical Association, 2013), also requires that consent be obtained for the use of any identifiable human data.

Privacy rights can be protected by depersonalising data, which is already a commonly implemented ethical practice. It is not typical for science to be interested in the identity attached to data; therefore, depersonalisation would rarely impact the potential scientific gain. At a minimum, depersonalisation can be achieved by removing obviously identifying information such as names or faces. Artificial Intelligence (AI) tools can also assist with this, as we have used in the present paper. However, it is important to consider that richer data sets may provide information that could be combined to identify a participant. For example, rich patient data, or even kinematic data that is only spatially or temporally based, could be backward engineered to identify the source. While this is unlikely, given that the motivation to do so would be low, it does pose a risk that should be carefully assessed.

Where a patient is concerned, consent, confidentiality, anonymity, and protection needs should be carefully considered from both an ethical and legal standpoint, as the data could be considered particularly sensitive. Video data of procedures need not always be added to a patient’s medical record if the video is collected solely for quality improvement, and the video would not be used to inform patient care (van Dalen et al., 2019).

### Moving toward a culture of data sharing

In sum, collective efforts toward accumulating skills performance data alongside relevant demographic or patient data have the potential to advance healthcare professional education substantially. In doing so, empirically validated competency targets can be established, and computer scientists can leverage the data to develop high-quality, robust tools to facilitate healthcare professionals and trainees to learn and maintain healthcare skills.

Establishing a culture of data sharing does face several challenges, although policies are rapidly evolving to support such initiatives. As policy is being developed globally, at a local level, healthcare and educational institutions may wish to establish their collaborative policies within current legal and ethical frameworks to advance research around healthcare professional skills education. In parallel, the educational and research community could benefit from establishing consensus on what may be considered informative data and guidelines for data organisation to facilitate access. By carefully considering the type of data required and its organisation, the field will balance the opportunity that exploratory work can bring with data minimisation principles. Established skills performance databases will then invite interdisciplinary researchers to engage with the institutions that have a stake in the outcomes (healthcare and educational institutions) to advance the field.

Here, we demonstrate the process in practice with an established interdisciplinary research team of healthcare professional educators, human movement scientists, and computer scientists. We provide a database of CPR skills and performances of varied experts who have been assessed for performance quality by two experts. Then, using computer vision and machine learning, we leverage this data to demonstrate the possibility of an Automatic Clinical Assessment tool for Basic Life Support.

### Automatic clinical assessment for basic life support

Early recognition of a cardiac event and quick application of CPR with high-quality chest compressions is advocated internationally (Berg et al., 2023; Merchant et al., 2020; Olasveengen et al., 2021; Resuscitation Council UK, 2021). Indeed, there is consensus within the evidence that high-quality CPR improves outcomes for patients in cardiac arrest (Gates et al., 2015). Thus, basic life support (BLS) education is essential to healthcare professional education. It is also a primary feature of first aid training delivered to individuals who are not healthcare professionals or trainees. High-quality chest compressions are reflected in hand and elbow position, compression depth, rate and recoil, alongside consideration of the angle of compression force application and rescuer safety (Resuscitation Council UK, 2021). Feedback during training has been found to significantly enhance compression quality (Baldi et al., 2017). Studies using Kinect depth cameras and pose estimation techniques show promising tracking and feedback provision capabilities (Lins et al., 2019; Xie et al., 2020). Indeed, real-time feedback from Kinect can significantly improve chest compression quality for rescuers who weigh below 71 kg (Wang et al., 2018). The body weight limit here may be attributed to a higher quality baseline in those with higher body weights. However, such a limit also indicates the importance of accessing diverse datasets. Other Kinect-based studies have demonstrated comparable benefits to real-time visual feedback for skills development in CPR (Semeraro et al., 2013). Although these Kinect-based studies demonstrate the utility of providing feedback for training purposes, Kinect does require specialist cameras and sensors, which may limit uptake.

Pose estimation can be performed using a computer and any camera. Initial work comparing expert ratings and evaluations from such computer vision techniques has shown promising results. In a study of arm angle (and chest-to-chest distance between team members), pose estimation was thought to be more precise in estimating arm angle than experts (Weiss et al., 2023). Here, we seek to demonstrate how deep learning techniques can provide an automatic assessment of CPR technique against a comprehensive set of metrics that assess both the quality of movement concerning the CPR performance and the postural safety of the performer.

Alongside this paper, we provide a CPR performance data set comprising a range of competencies for use to advance research that understands technical competency and builds tools to support the development of such competencies. The data set includes demographic information, self-ratings of confidence and frequency of performance, and two expert evaluations of the performance. The database contains video data of CPR from multiple angles with a checkerboard allowing for 3D reconstruction.

## Methods

### Participants

Participants were recruited on three different days. Participants were recruited from Northumbria University’s Department of Nursing and Midwifery on the first day via the researchers’ networks. Recruitment resulted in 22 participants with varied expertise, ranging from complete novices who had never performed CPR before to individuals with extremely high levels of expertise in CPR (trained professionals and educators who regularly perform CPR). On the second day, 20 students who attended a skills event held by the Department were recruited on a voluntary basis as an opportunity to practice CPR and contribute to research. They were of varied skill levels, with some having previously undergone training and others not; all were students of the Department. On the third day, the recording session was set up to coincide with a first-year training session; 10 first-year students in the Department of Nursing and Midwifery and one non-student in the Department were recruited. Thus, data from 53 participants was collected. Authors [MDC, FXZ, TC, DM, JR, CF, LJP, AP] participated. All participants provided informed consent and indicated how they would like their data to be used (Video or evaluative data available only to the research team/available in a safeguarded science repository for scientific use). For the present paper, we have used data from all participants; where participants consented (n = 40) videos with faces digitally obscured have been uploaded to UK Data Service Reshare (Constable et al, 2024b). Participants who did not consent to their data being used outside the research team have not been included in the repository. Researchers or educational professionals may access the repository in a safeguarded manner subject to adhering to the terms and conditions of the repository. To gain access researchers must email the data controller (MDC) stating their status as a researcher and their intention for the data; they will then be granted access. Northumbria’s Ethics System (No. 44602) approved the research, and all research was performed per the Declaration of Helsinki.

The average age of participants was 33.60 years (SD = 13.00); and 14 were men, and 39 were women (self-declared), see Table 1 for age by gender. Participants self-reported confidence in performing CPR, ranging the full spectrum of possible responses from Very Confident to Very Unconfident (5-point Likert scale), with the median response being ‘Somewhat confident’. Self-reported frequency also ranged the full spectrum of possible responses from Very Frequently to Very Infrequently (5-point Likert scale), with the data being skewed toward infrequent performance (median response = Very Infrequently). The skew in the data reflects that both students and clinicians use CPR skills relatively infrequently and thus require regular refresher training (Oermann et al., 2011).

**Table 1:** Average age (Standard deviation in parentheses) by self-identified gender

Gender	Male	Female
Age	42.57(15.02)	30.38 (10.67)

### Data protection

We obtained informed consent from participants who were able to indicate how they would like their data to be used and stored. Further, the data has been depersonalised by digitally obscuring their faces. Access is granted with safeguarding protections such that users must agree to the terms and conditions of the repository. Importantly, these terms and conditions require users to be registered, to only use the data for research or learning purposes, and to maintain the confidentiality of the participants. Any participants who indicated that they did not want their data to be used outside of the research team have not been included in the data set available to researchers external to the research team. Furthermore, participants could elect to have only their videos or evaluations shared should they wish.

### Recordings

**Fig. 1:**
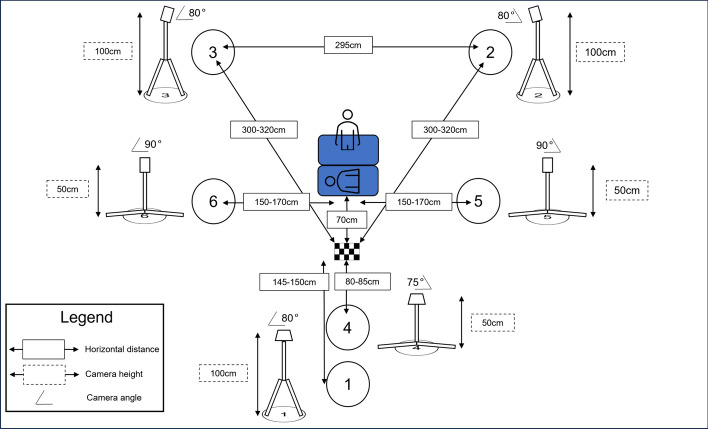
The recording space. Circles depict the location of the cameras. The checkerboard was placed in front of the task space with one foam mat for the manikin and one foam mat for the participant. Approximate distances between cameras are provided, although there was some slight variation between days

Each person was recorded using 6 Go-Pro Cameras. The first camera was set up to have a wide frontal view (see Fig. 1). Cameras 2 and 3 were placed behind the participant, offset to the right and left. Camera 4 was placed in front of Camera 1 to provide a lower and closer frontal view. Cameras 5 and 6 were placed perpendicularly to the direction the participant was facing in line with the participant. A checkerboard was placed in front of a QCPR manikin and in view of all six cameras as a common landmark. The CPR space was defined for the participant with two foam mats. One for them to kneel on, the manikin was placed on the other.

Participants were asked to perform 4 sets of 30 chest compressions for each recording with a short pause in between to rest. Participants were asked at the beginning and end of the task to clap. This clap was used to calibrate cameras in time.

### Ratings

An evaluative framework (see supplementary material) was developed in consultation with the BLS experts on the team (TC, DM). Both experts have been teaching BLS for over 20 years in clinical and educational settings, and the UK Resus Council recognises both as Advanced Life Support Instructors, representing exceptional expertise in the field. Given that the present data was collected within the UK educational system, the evaluative dimensions were informed by guidelines from the Resuscitation Council UK (2021). Additional evaluative dimensions were included to reflect good posture and technique taught to maintain endurance, reduce the likelihood of injury, and prevent fatigue. Each evaluative dimension represented a factor that would be currently instructed in the educational setting; nevertheless, in practice, each factor is not equally important for patient outcomes as indicated by the International Liaison Committee on Resuscitation’s recommendations, which are updated yearly based on cumulative science (Berg et al., 2023).

For each cycle (4 per participant), one point was provided for good form in the following evaluative dimensions: Hand Position (Centre of the chest, within one average hand margin), Arm Position (Straight Arms, minimal flex in elbows or minimal variability in elbow joint angle), Shoulder Position (Over patient, the line from centre of patient to shoulders should be perpendicular), Depth of Compressions (5-6cm), Rate of Compressions (100–120 per minute), Release (complete recoil of chest, hands return to neutral start point). A metronome was used to assist in evaluating the rate of compressions. Experts also coded for incorrect form (see supplementary material – evaluative checklist), such that if there were multiple ways a participant could exhibit poor form, that will be reflected in the data (e.g. Depth of compressions could be either too shallow or too deep). Overall ratings (Excellent, Good, Borderline, Poor, Unacceptable) for each cycle were also provided.

The two expert raters initially rated alone and then resolved any discrepancies to provide an agreed rating. To determine rater agreement when raters were rating alone we calculated weighted Cohen’s kappa for overall ratings for each cycle (rated: Unacceptable, Poor, Borderline, Good, Excellent). Overall, raters were in moderate agreement for Cycles 2, 3, and 4 when they rated alone, κs = 0.550, 0.567, 0.518, respectively. Agreement was poor for Cycle 1, κs = 0.204, potentially reflecting inconsistencies in performance during the initial cycle, which could reflect a ‘practice’ run.

### Automatic clinical assessment

**Fig. 2:**
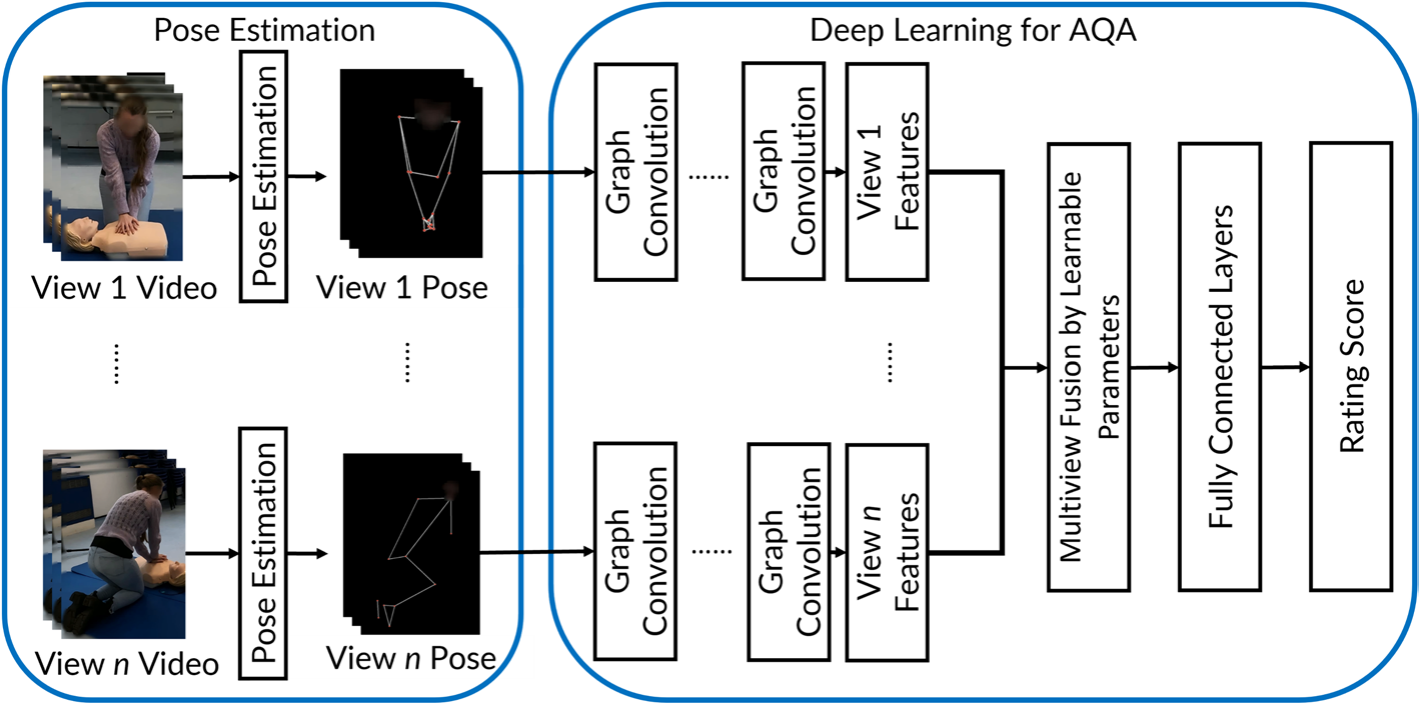
Overview of the framework for our automatic AQA. The process begins with extracting joint motion information from raw video footage captured from multiple viewpoints, using pose estimation techniques. Following this, deep learning algorithms analyse the spatial–temporal features of the extracted data from each angle. The system then integrates these features across different views to accurately predict the performance ratings for each assessed skill

Our methodology systematically assesses CPR techniques using human motion data, mirroring expert evaluations while leveraging the advantages of automation. Our approach to assessing clinical techniques includes two main components: markerless pose estimation and a deep learning network designed specifically for Automatic Action Quality Assessment (AQA), as shown in Fig. 2. The first step in our framework is to use markerless pose estimation to capture the 3D positions of a participant’s joints from different angles in the video. This process is notable because it does not rely on physical sensors or markers attached to the participant. Instead, it directly analyses the video frames to identify and track the movements of the joints. Following this, the pose information that has been extracted is input into a deep learning network. This network is trained to assess the quality of CPR performance against predefined criteria as same as those used in manual expert assessments. The network produces ratings for various aspects of the CPR technique, thus providing an objective, automated evaluation of the participant’s skill level.

## Markerless pose estimation

**Fig. 3:**
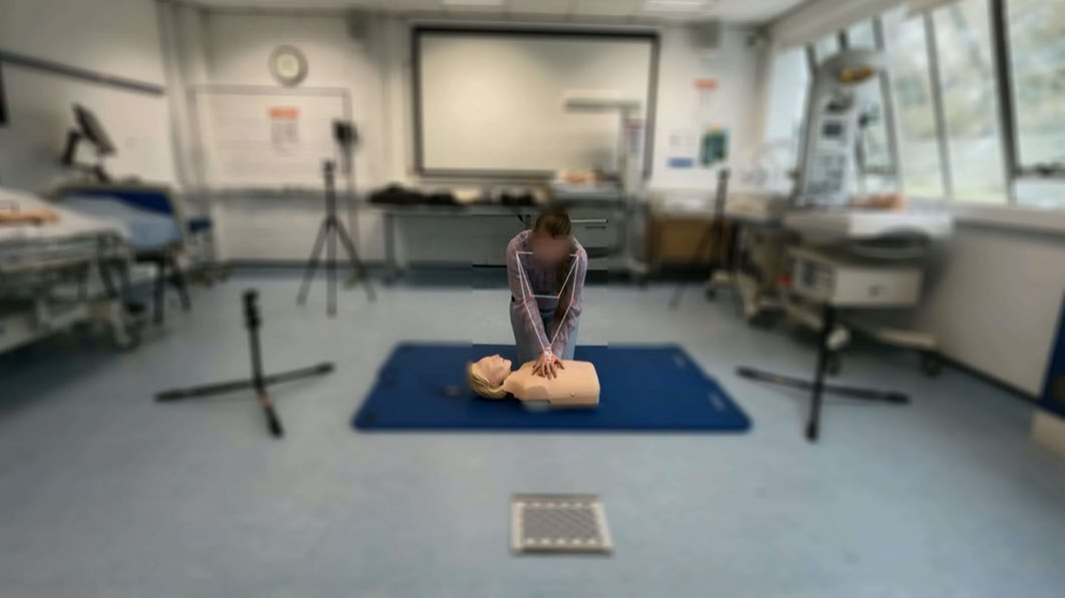
An example of the estimation visualisation. Key joint landmarks relevant to the clinical technique are clearly captured

We employed MediaPipe (Lugaresi et al., 2019), a framework that enables machine learning models to interpret and analyse human motion video data. This approach allowed us to capture the movements of various joints, outputting their positions in a three-dimensional space ($X$, $Y$, and $Z$ coordinates) relative to a standard’ world coordinate system,’ which provides a consistent frame of reference for movement analysis. The results of our pose estimation are organised in a structured format, denoted as ${P}_{k}\in {R}^{T\times N\times C}$, where $k$ denotes the camera viewpoint, $T$ denotes the total number of video frames (or the duration of the video), $N$ denotes the count of distinct joints tracked, and $C$ denotes various data features for each joint, including their spatial coordinates and the confidence level of these estimations. An illustrative example of how we visualise this pose estimation data can be seen in Fig. 3.

### Deep learning for automatic action quality assessment

#### The graph representation of human skeleton

After obtaining the pose estimation outputs, denoted as${P}_{k}$, which detail the positions of various joints over time, we conceptualise the human skeleton as a graph structure. This graph-based representation, $\left(V, E\right)$, allows our neural network to incorporate the anatomical and biomechanical constraints inherent to human movement (Feng et al., 2022). In this graph, the set of nodes $V$ represents the joints, indexed from 1 to$N$, where each node ${v}_{i}$ corresponds to a specific joint. The edges$E$, represent the connections (i.e. bones) between these joints, such as bones or ligaments, defined within a set $S$ that specifies which pairs of joints are connected, which promotes the network to represent the physical structure of the human body.

#### The multiview spatial temporal graph convolutional network

After defining the graph representation based on the pose estimation results, we developed a multiview neural network tailored for Automatic Action Quality Assessment (AQA), using the estimated human poses as its foundation. To achieve this, we adopted Spatial Temporal Graph Convolutional Networks (STGCN) (Yan et al., 2018), configuring the network with a five-layer architecture to robustly capture and model the dynamics of the pose estimation data from each camera viewpoint. This approach incorporates the skeleton graph structure dynamics of the human body to constrain our deep learning model for joint motion analysis. The detailed operation for the input pose estimation ${\text{P}}_{k}$ as follow:

$${H}_{k}= {\wedge }^{-1/2}A{\wedge }^{-1/2}{P}_{k}{W}_{k},$$

where ${H}_{k}$ denotes the learned hidden feature for camera viewpoint $k$, ${W}_{k}$ denotes the learnable parameters in our STGCN. $A$ and $\wedge$ denote the adjacency matrix and its normalised form, respectively. The value of $A$ is defined such that ${A}_{i,j}=1$ if ${v}_{i}{v}_{j}\in E$, which introduces the semantics of our defined human skeleton graph into deep learning.

Then, the learned features ${H}_{k}$ from each view are then fused via a learnable parameter. Finally, a two-layer fully connected neural network is applied to estimate the final rating for each item score related to clinical technique quality.

### Optimisation

In the optimisation phase, our primary aim is to enhance the accuracy of our network’s predictions. To this end, we utilise the Mean Absolute Error (MAE) as our metric of choice. MAE is a straightforward yet effective measure that calculates the average of the absolute differences between the predicted values by our network and the rating values (‘ground truth’) after clinical experts’ agreement. This metric is particularly useful for our item scores, as it clearly indicates how close our predictions are to reality, on average, without being influenced by the direction of errors. The formula for our loss function, which incorporates MAE, is given by:

$$L= \sum_{q=1}^{Q}|\widehat{{y}_{q}}-{y}_{q}|$$

where $Q$ denotes the different item scores related to clinical technique quality, $\widehat{{y}_{q}}$ and ${y}_{q}$ denote the predicted score and the ground truth score for each item, respectively. Our goal during training is to minimise this loss, which means reducing the average absolute error between our predictions and the expert ratings, thereby aligning our network’s assessments more closely with the expert evaluations.

For the optimisation process, we employ the Adam optimiser, a widely used optimisation algorithm known for its effectiveness in handling sparse gradients and automatically adjusting the learning rate. This choice promotes a more efficient and robust training process, with an initial learning rate set at 0.01 and a weight decay of 0.1, to gradually improve our model’s performance by iteratively adjusting its parameters in a direction that minimises the loss function.

### Evaluation

We evaluate our automatic AQA framework’s performance using Mean Absolute Error (MAE), employing a fivefold cross-validation approach. In each validation cycle, 80% of the data was used as the training set, while the remaining 20% was used as the test set, ensuring different train and test sets for each iteration. The training epoch is set to 100. Our evaluation not only compares our automated method’s predictions to the final expert-agreed scores but also examines the alignment of initial individual expert annotations with these consensus ratings.

The comparison involves calculating the MAE between our method’s predictions and the expert-agreed scores and, similarly, between individual expert scores before consensus and the final agreed scores. This approach highlights our method’s potential accuracy in relation to initial expert assessments. MAE was selected as the primary metric due to its interpretability, robustness, and consistency. It is a common metric for skill assessment in the biomedical engineering domain (Anastasiou et al., 2023; Wagner et al., 2023) It allows us to directly quantify the average error in our model’s predictions compared to expert ratings. Thus, we use MAE across both optimisation and evaluation phases, facilitating a clearer comparison of our model’s accuracy relative to expert assessments.

For a fair comparison, we align our automated assessments with expert evaluations by focusing on data from cameras 1, 4, and 5, which the experts predominantly used. This strategy ensures that our method is evaluated from the most relevant perspectives for accurate clinical technique assessment. Cycle 1 was included in the analysis to assess the performance of our system in scenarios where human raters have difficulty reaching consensus. The low agreement among experts in Cycle 1 highlights the complexity of certain CPR assessments and underscores the importance of having an automated system that can provide consistent evaluations. By including Cycle 1, we ensure that our system is tested not only on straightforward cases where human agreement is high but also on more challenging cases where human agreement is low.

We implemented our method with PyTorch 1.10.1 and trained the models using one Nvidia GeForce GTX 2080 Ti GPU. For further reproduction and implementation of our research, our code and step-by-step deployment instructions can be found on our GitHub page: https://github.com/FrancisXZhang/CPR.

## Results and discussion of AQA

In our analysis, Table 1 illustrates the MAE comparisons between the automatic AQA framework and the initial scores given by two human evaluators. Specifically, the MAE values represent the average difference between the scores assigned by our AQA system or each evaluator and the final agreed-upon ground truth scores established through expert consensus. The full score for each evaluated item is 4. In most cases, the error margin of our framework remains below 1, underscoring our automated methods’ potential accuracy and applicability. When comparing our method with manual assessments, we found that our automatic AQA consistently exhibits significantly lower error in evaluating hand, arm, and shoulder positions. This may be attributed to our framework’s reliance on precise pose information, providing a more objective assessment of the participant’s posture. Our AQA exhibited higher error rates in the compression depth and compression rate items. This discrepancy could be because these two items require assessing interactions between the participant and the dummy (Kılıç et al., 2018), something our framework currently does not capture. This demonstration focused solely on the pose information of the participant and did not incorporate visual interaction data between humans and objects; further work could establish the importance of considering such interactions. It is also noteworthy that the expert raters used a metronome to assist in their rate judgements, which may account for a higher than typical expert-assessed accuracy rate in this dimension (Table 2).

**Table 2:** Mean average error (Human experts vs. AQA framework)

Item	Evaluator 1	Evaluator 2	AQA
Hand Position	1.62	1.08	0.33
Arm Position	0.70	0.15	0.07
Shoulder Position	0.40	0.34	0.13
Depth	0.49	0.30	0.69
Rate	0.89	0.11	1.67
Compression Release	1.04	0.98	1.00
Total	3.96	2.69	2.98

We acknowledge that the size of our dataset, comprising 53 samples, might appear limited for training deep learning models. However, we employed a fivefold cross-validation approach, promoting robust evaluation by using 80% of the data for training and 20% for testing in each fold, which helps in assessing the model’s performance comprehensively and mitigating overfitting. Our dataset size is comparable to those used in other research within the domain of healthcare training systems, such as the 10 cases used in Liao et al. (2020) demonstrating the feasibility of using similar dataset sizes. Moreover, the results indicate that the tool performs well as compared to human raters, confirming the reliability of the measure.

**Fig. 4:**
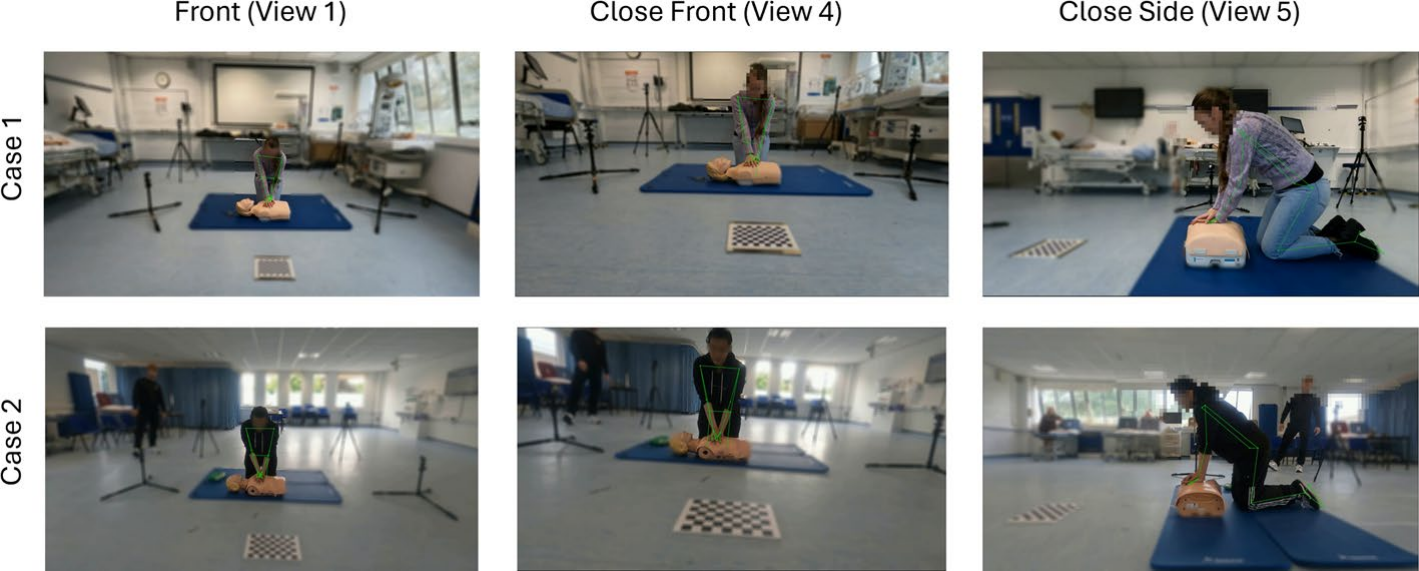
Qualitative exemplars for comparing our model with human raters. Our model’s performance aligns with the final rating after agreement, whether in the human raters’ initial agreed score or their conflicted score

To further compare our model with the human raters, qualitative research for individuals by our model compared to the raters’ evaluations is shown in Fig. 4. To better demonstrate the efficiency of our work, we included both cases of conflicts and agreements in our exemplars. As the main potential advantage of our system is based on pose estimation, these exemplars are mainly focused on pose-related scores.

In Fig. 4, Case 1, both human raters gave high scores for the subject’s hand, arm, and shoulder positions (score from human raters = 4, maximum rating = 4). Similarly, our model also gave a score of 4 for these pose-related aspects. In Fig. 4, Case 2, although the reviewers gave a score of 4 for the arm and shoulder pose of the participant, one rater gave a 0 for the hand position while the other gave a 4 before they reached an agreement. The reason for the 0 was ‘Too far toward the feet’, but the rater who gave a 4 thought it was ‘Just in the center of the chest’. After double-checking the video, they ultimately agreed to give the subject a score of 4, as the hand pose was, indeed, ‘In the center of the chest (within one average hand margin)’.

One major reason for this conflict is that the raters typically make their judgments based on a single camera view. However, the information from one camera can be limited due to different participants’ angles towards the camera and varying initial postures, which sometimes affects their judgment. For instance, in Case 2, the ratings are highly focused on the close front view. The participant’s habit of performing CPR vertically makes their hand compression position appear farther from their body for the human raters. Our method, which fuses information from multiple camera views, overcomes this limitation. Even though our pose estimation may occasionally show misdetections (pose estimation visualisation for the full video can be found in the Supplementary Material), summarising the motion information from multiple views makes our rating results more robust for pose-related scores.

The present work sought to illustrate how accumulating skills performance datasets in real or simulated settings could provide a foundation for understanding healthcare skills and building educational technology to support healthcare professionals and educators. A fundamentally interdisciplinary approach (in this case, with a strong emphasis on computer science) in the study of technical skills may assist in ensuring healthcare professional competence. Specifically, we have shown how innovations in computer vision can be leveraged to provide (1) Data from real settings that can be scientifically assessed (e.g. the spatial location of each joint in cartesian coordinate space alongside estimation confidence) and (2) an assessment of performance quality based on both objective measurements and learned parameters from expert raters. This assessment technique demonstrated comparable accuracy in overall assessment relative to our expert raters, indicating the validity of the approach. Furthermore, the margin of error from this assessment technique was typically below 1, indicating that accuracy between defined thresholds of competency was excellent. However, specific to performance features, our assessment technique sometimes outperformed or underperformed relative to the raters. Our intention with this work is to call for foundations to be put in place that allow for the collection and sharing of diverse healthcare technical skills performances that provide the opportunity for interdisciplinary collaborations to enhance the efficacy and efficiency of healthcare professional education.

Manikins and simulators that provide automated feedback on a range of critical measures to facilitate the acquisition of an appropriate skill threshold exist (e.g. QCPR manikins (Laerdal)). However, they are often limited in the type of feedback they can provide, highly specific to a given skill or class of skills, or expensive. The computer vision approach demonstrated in the present work has the potential to provide automated and targeted feedback for a range of skills that can be assessed visually with low-cost video cameras and computers that would already be present within an educational setting. Furthermore, the fact that a range of skills could be assessed with the relatively low-cost set-up and without the need for specialist simulators represents an economic benefit. The flexibility of the computer vision approach also makes it ideal for assessing complex skills performance in highly realistic simulations. Indeed, self-training programmes exist using items that can be found around the home for a makeshift manikin (Wanner et al., 2016), it is possible that this technology could be implemented using a webcam to provide feedback to the trainee at home.

In future research, we plan to consider human-object interaction in our model. Incorporating interactions with other humans or objects into our model necessitates a comprehensive approach. First, we would expand our dataset to include scenarios involving human–human and human-object interactions, ensuring a wide range of contexts and activities. Second, more detailed annotations would be required to label these interactions accurately, such as the actual physical contact between humans and objects to make training closer to real-world conditions (Zhou et al., 2023). Third, our model architecture would need modifications to handle the additional complexity, such as integrating temporal-based pose estimation for more consistent motion information capture (Zhou et al., 2023). By addressing these steps, we aim to significantly enhance the model’s utility in more realistic and dynamic environments, ultimately improving its applicability for various educational and training purposes.

## Supplementary Information

Below is the link to the electronic supplementary material.

Supplementary file1 (PDF 988 KB)

Supplementary file2 (MP4 103627 KB)

Supplementary file3 (MP4 118570 KB)

Supplementary file4 (MP4 169603 KB)

Supplementary file5 (MP4 115344 KB)

Supplementary file6 (MP4 187673 KB)

Supplementary file7 (MP4 310008 KB)

## Data Availability

Participants provided consent as to what data they wished to have shared (Evaluation & Demographic Data/Video Data). Data for which participants provided consent will be made available in safeguarded and anonymised form via UK Data Service’s ReShare Repository. This data can be found here: Constable, M. D., Zhang, F. X., Connor, T., Monk, D., Rajsic, J., Ford, C., Park, L. J., Barker, S., Platt, A., Porteous, D., Grierson, L., & Shum, H. P. H. (2024). Cardiopulmonary Resuscitation Performance: Video, Demographic and Evaluation Data, 2023 [Data Collection]. UK Data Service. https://doi.org/10.5255/UKDA-SN-857038.
