# Supplementary material for: Advancing healthcare practice and education via data sharing: demonstrating the utility of open data by training an artificial intelligence model to assess cardiopulmonary resuscitation skills
Source: Adv Health Sci Educ Theory Pract. 2024 Sep 9;30(1):15–35. doi: 10.1007/s10459-024-10369-5 (PMC11926039; doi:10.1007/s10459-024-10369-5)
Supplement: Supplementary file 1 — Supplementary file1 (PDF 988 KB) [file 10459_2024_10369_MOESM1_ESM.pdf]

BLS Ratings form

* Required

* This form will record your name, please fill your name.

Which participant are you rating?

1. Which participant are you rating? *

   First digits of video filename (the participant number) If joint rating please type 'J' in front.

2. What camera angles did you use? *

- [ ] Camera 1 (front wide)
- [ ] Camera 2 (back left)
- [ ] Camera 3 (back right)
- [ ] Camera 4 (front close)
- [ ] Camera 5 (side left)
- [ ] Camera 6 (side right)

Cycle 1 ratings

3. Cycle 1 - Hand Position: * (1 Point)

- [ ] Centre of chest (within one average hand margin)
- [ ] Too far left
- [ ] Too far right
- [ ] Too far toward the head
- [ ] Too far toward the feet

4. Cycle 1 - Do you have any extra comments on the hand position?

5. Cycle 1 - Arm Position * (1 Point)

- ◯ Straight Arms (minimal flex in elbows or minimal variability in the elbow joint angle)
- ◯ Bent arms

6. Cycle 1 - Do you have any extra comments on the arm position?

7. Cycle 1 - Shoulder position * (1 Point)

- ◯ Over patient (line from centre of patient to shoulders perpendicular)
- ◯ Not directly over compression site

8. Cycle 1 - Do you have any extra comments on the shoulder position?

9. Cycle 1 - Depth of Compressions * (1 Point)

- ◯ 5-6 cms
- ◯ Too shallow
- ◯ Too deep

10. Cycle 1 - Do you have any extra comments on the depth of compressions?

11. Cycle 1 - Rate of Compressions * (1 Point)

- ◯ 100-120 p/m
- ◯ Too slow
- ◯ Too fast

12. Cycle 1 - Do you have any extra comments on the rate of compression?

13. Cycle 1 - Correct release * (1 Point)

- ◯ Complete recoil of chest hands return to neutral start point)
- ◯ Incomplete recoil of chest

14. Cycle 1 - Do you have any extra comments on the release?

15. Cycle 1 - Overall rating *

- ◯ Excellent
- ◯ Good
- ◯ Borderline
- ◯ Poor
- ◯ Unacceptable

16. Cycle 1 - Do you have any extra comments on Cycle 1?

Cycle 2 ratings

17. Cycle 2 - Hand Position: * (1 Point)

- [ ] Centre of chest (within one average hand margin)
- [ ] Too far left
- [ ] Too far right
- [ ] Too far toward the head
- [ ] Too far toward the feet

18. Cycle 2 - Do you have any extra comments on the hand position?

19. Cycle 2 - Arm Position * (1 Point)

- ◯ Straight Arms (minimal flex in elbows or minimal variability in the elbow joint angle)
- ◯ Bent arms

20. Cycle 2 - Do you have any extra comments on the arm position?

21. Cycle 2 - Shoulder position * (1 Point)

- ◯ Over patient (line from centre of patient to shoulders perpendicular)
- ◯ Not directly over compression site

22. Cycle 2 - Do you have any extra comments on the shoulder position?

23. Cycle 2 - Depth of Compressions * (1 Point)

- ◯ 5-6 cms
- ◯ Too shallow
- ◯ Too deep

24. Cycle 2 - Do you have any extra comments on the depth of compressions?

25. Cycle 2 - Rate of Compressions * (1 Point)

- ◯ 100-120 p/m
- ◯ Too slow
- ◯ Too fast

26. Cycle 2 - Do you have any extra comments on the rate of compression?

27. Cycle 2 - Correct release * (1 Point)

- ◯ Complete recoil of chest hands return to neutral start point)
- ◯ Incomplete recoil of chest

28. Cycle 2 - Do you have any extra comments on the release?

29. Cycle 2 - Overall rating *

- ◯ Excellent
- ◯ Good
- ◯ Borderline
- ◯ Poor
- ◯ Unacceptable

30. Cycle 2 - Do you have any extra comments on Cycle 2?

Cycle 3 ratings

31. Cycle 3 - Hand Position: * (1 Point)

- ☐ Centre of chest (within one average hand margin)
- ☐ Too far left
- ☐ Too far right
- ☐ Too far toward the head
- ☐ Too far toward the feet

32. Cycle 3 - Do you have any extra comments on the hand position?

33. Cycle 3 - Arm Position * (1 Point)

- ○ Straight Arms (minimal flex in elbows or minimal variability in the elbow joint angle)
- ○ Bent arms

34. Cycle 3 - Do you have any extra comments on the arm position?

35. Cycle 3 - Shoulder position * (1 Point)

- ◯ Over patient (line from centre of patient to shoulders perpendicular)
- ◯ Not directly over compression site

36. Cycle 3 - Do you have any extra comments on the shoulder position?

37. Cycle 3 - Depth of Compressions * (1 Point)

- ◯ 5-6 cms
- ◯ Too shallow
- ◯ Too deep

38. Cycle 3 - Do you have any extra comments on the depth of compressions?

39. Cycle 3 - Rate of Compressions * (1 Point)

- ◯ 100-120 p/m
- ◯ Too slow
- ◯ Too fast

40. Cycle 3 - Do you have any extra comments on the rate of compression?

41. Cycle 3 - Correct release * (1 Point)

- ◯ Complete recoil of chest hands return to neutral start point)
- ◯ Incomplete recoil of chest

42. Cycle 3 - Do you have any extra comments on the release?

43. Cycle 3 - Overall rating *

- ◯ Excellent
- ◯ Good
- ◯ Borderline
- ◯ Poor
- ◯ Unacceptable

44. Cycle 3 - Do you have any extra comments on Cycle 3?

Cycle 4 ratings

45. Cycle 4 - Hand Position: * (1 Point)

- [ ] Centre of chest (within one average hand margin)
- [ ] Too far left
- [ ] Too far right
- [ ] Too far toward the head
- [ ] Too far toward the feet

46. Cycle 4 - Do you have any extra comments on the hand position?

47. Cycle 4 - Arm Position * (1 Point)

- ◯ Straight Arms (minimal flex in elbows or minimal variability in the elbow joint angle)
- ◯ Bent arms

48. Cycle 4 - Do you have any extra comments on the arm position?

49. Cycle 4 - Shoulder position * (1 Point)

- ◯ Over patient (line from centre of patient to shoulders perpendicular)
- ◯ Not directly over compression site

50. Cycle 4 - Do you have any extra comments on the shoulder position?

51. Cycle 4 - Depth of Compressions * (1 Point)

- ◯ 5-6 cms
- ◯ Too shallow
- ◯ Too deep

52. Cycle 4 - Do you have any extra comments on the depth of compressions?

53. Cycle 4 - Rate of Compressions * (1 Point)

- ◯ 100-120 p/m
- ◯ Too slow
- ◯ Too fast

54. Cycle 4 - Do you have any extra comments on the rate of compression?

55. Cycle 4 - Correct release * (1 Point)

- ○ Complete recoil of chest hands return to neutral start point)
- ○ Incomplete recoil of chest

56. Cycle 4 - Do you have any extra comments on the release?

57. Cycle 4 - Overall rating *

- ○ Excellent
- ○ Good
- ○ Borderline
- ○ Poor
- ○ Unacceptable

58. Cycle 4 - Do you have any extra comments on Cycle 4?

Overall rating

59. All Cycles - Overall rating *

- Excellent
- Good
- Borderline
- Poor
- Unacceptable

60. Any extra comments on all cycles/overall performance?

This content is neither created nor endorsed by Microsoft. The data you submit will be sent to the form owner.

Microsoft Forms
